# Repair of distal finger soft-tissue defects with free fibular great toe neurovascular flaps

**DOI:** 10.1186/s12891-024-07563-2

**Published:** 2024-06-18

**Authors:** Fengnian Yu, Fen Xiao, Guorui Peng, Gang Lin, Wensong Wang, Chao Xie, Lijun Lin

**Affiliations:** 1 Department of Orthopedics, Jiangmen People’s Hospital, Jiangmen, 529020 Guangdong P. R. China; 2 Department of Joint and Orthopedics, Zhujiang Hospital, Southern Medical University, Guangzhou, 510280 Guangdong P. R. China; 3 Department of Orthopedics, Guzhen People’s Hospital, Zhongshan, 528421 Guangdong P.R. China

**Keywords:** Free flap with digital nerve, Finger pulp defect, Fingerprint reconstruction, Biological identification, Repair pattern

## Abstract

**Background:**

This work aimed to investigate the change in fingerprint depth and the recovery rule of fingerprint biological recognition function after repairing finger abdominal defects and rebuilding fingerprint with a free flap.

**Method:**

From April 2018 to March 2023, we collected a total of 43 cases of repairing finger pulp defects using the free flap of the fibular side of the great toe with the digital nerve. After surgery, irregular follow-up visits were conducted to observe fingerprint clarity, perform the ninhydrin test or detect visible sweating with the naked eye. We recorded fingerprint clarity, nail shape, two-point discrimination, cold perception, warm perception and fingerprint recognition using smartphones. The reconstruction process of the repaired finger was recorded to understand the changes in various observation indicators and their relationship with the depth of the fingerprint. The correlation between fingerprint depth and neural repair was determined, and the process of fingerprint biological recognition function repair was elucidated.

**Result:**

All flaps survived, and we observed various manifestations in different stages of nerve recovery. The reconstructed fingerprint had a clear fuzzy process, and the depth changes of the fingerprint were consistent with the changes in the biological recognition function curve.

**Conclusion:**

The free flap with the digital nerve is used to repair finger pulp defects. The reconstructed fingerprint has a biological recognition function, and the depth of the fingerprint is correlated with the process of nerve repair. The fingerprint morphology has a dynamic recovery process, and it can reach a stable state after 6–8 months.

**Supplementary Information:**

The online version contains supplementary material available at 10.1186/s12891-024-07563-2.

## Introduction

Fingers are vital parts for performing the functions of what the hand normally does. As the popularity of fingerprinting equipment is increasing, fingerprint use for personal identification has become integrated in numerous scenarios, such as access to an individual’s telephone. Unfortunately, distal finger soft-tissue defects may result in much inconvenience [1]. Multiple surgical strategies (e.g. Matriderm dermal substitute, interdigital artery flaps, second-layer palmar graft and toe flaps) have been introduced for repair purposes, but the common treatments remain controversial, and the repair of defects remains challenging [2–4]. 

The skins over the fibular side of the great toe and the distal finger possess similar characteristics; both are hairless, thick and have a high density of nerve endings and eccrine sweat glands [5, 6]. Several studies have shown that this surgical strategy has advantages in terms of minor donor site morbidity, ideal form and nerve recovery [7–9]. However, few studies reported postoperative fingerprint recognition, and studies focusing on recovery at different times are scarce. This study aimed to fill the research gap. A total of 43 patients who underwent transplantation of neurovascular flaps from the free fibular region to the great toe because of distal finger soft-tissue defects in our medical institutions were identified. Patients were divided into six groups by intervals between the operation date and the test date. The postoperative fingerprint register and recognition were the leading targets. We also noted four other parameters: warm sense, cold sense, static two-point discrimination and ninhydrin staining.

## Materials and methods

This study was guided by the Declaration of Helsinki. The Medical Ethics Committee of Guzhen People’s Hospital approved this study (Approval ID: GZLLPS 20180109-01). The candidates provided written informed consent. Tests were performed by two surgeons. When the two came to the same result, this was recorded. Once divergence occurred, another surgeon was asked for feedback. Before the tests, surgeons communicated with candidates to build trust, explain the test process and teach candidates to discriminate stimulation. The following points were noted during testing: (1) Tests should be carried out in a quiet, peaceful and comfortable environment; (2) Before testing, surgeons and candidates should wash and dry their hands. (3) We repeated all tests three times. When the three results were the same, they were recorded. Once divergence occurred, the result that appeared twice was recorded. (4) One test could last 3 s. The interval between the two tests was at least 5 s.

### Inclusion and exclusion criteria

Inclusion criteria were as follows: (1) patients were younger than 60 years old; (2) crush injury to the distal fingers; and (3) patients were repaired with a free flap of the fibular side of the toe with nerve anastomosis. The blood supply vessel was the fibular side artery of the toe base. During the surgery, attention should be paid to reconstructing the fingerprint.

Exclusion criteria were as follows: (1) patients suffered from chronic diseases that affect nerve growth, like diabetes; and (2) patients had phalanx fractures or bones missing.

### Grouping

The growth rate of axons during nerve regeneration varies depending on the nature of the nerve and injury. About 8 days after nerve injury, the regenerated axons begin to slowly form myelin sheaths from near to far, with a slow increase in thickness that takes about a year to complete [10, 11]. Therefore, the evaluation time for peripheral nerve regeneration should be based on different experimental purposes, nerves and injury types. If the maturity and functional recovery level of regenerated nerve fibres is evaluated, a long observation endpoint is generally selected, ranging from 3 months to more than 1 year. In general, observation is conducted at intervals of 1 month in the early postoperative period, followed by intervals of 3–6 months in the middle and late stages [12–14]. In this study, 43 patients were separated into six groups according to intervals between the operation date and the survey date: the 0–35 days group, the 36–75 days group, the 76–105 days group, the 106–215 days group, the 215–365 days group and the over 365 days group.

### Surgical procedure and postoperative care

The same surgical team carried out all operations. The structural separation and anastomoses were performed under the microscope. The surgical procedure utilised in this study was designed based on our extensive experience and previous surgical outcomes, which were published in several Chinese medical journals. These prior studies have provided us with insights into flap reconstruction techniques, which have been instrumental in refining the current surgical approach.

According to the condition of defects, the patterns of flaps were designed at the fibular aspect of the ipsilateral or contralateral great toes by the same experienced surgeon. Compared with the diameter of defects, that of the flap was 2–3 mm larger. The expected incision did not extend beyond the centre line of the plantar. A stripe of leather was devised to prevent over-tightened suture on the pedicle.

Brachial plexus block anaesthesia was performed. A tourniquet was tied around the proximal upper arm that was badly injured. Debridement was carried out. The proper palmar digital arteries and nerves were identified. One or two veins with wide diameters were marked. We measured distances between structures.

A tourniquet was tied around the proximal thigh. Combined spinal and epidural anaesthesia was carried out. We made incisions at the proximal acrotarsium, moving to the sides; one or two veins connecting to flaps were found and intercepted. We made incisions at the web areas, moving to the sole, and the proper digital arteries and nerves were detected. The flap was peeled completely. Thin soft tissue was left to cover the surface of the phalanx. We ensured arteries and nerves were kept intact in flaps. The excess fat was removed.

We analysed the corresponding structures between defects and flaps. We made sure that the toeprints were aligned with the fingerprints.

After surgery, patients were treated with antibiotics, anticoagulants, antispasmodics and drugs to promote nerve growth activity. Visible light illumination was applied to keep it warm. Under the guidance of surgeons, rehabilitation began within 7 days. Stitches were removed at 13–14 days after surgery.

### Fingerprint registers and recognition

The results of the fingerprint register and recognition were achieved by one phone. If the candidates had registered repaired fingerprints in the database and had used their repaired fingers to unlock the phone within five attempts, the result would have been marked as (1) If not, the result would have been marked as 0. Red printing oil was used to record fingerprint shapes. We set clarity ambiguity or missing record as 0, relatively clear as 1 and very clear as (2) The definition of relevant standards was as follows:

Relatively Clear: This descriptor is used when the fingerprint pattern is distinct, but certain ridge details may be slightly blurred or less distinct The overall pattern allows for a general identification, yet it may not meet the highest standards of claim required for some forms of biological analysis.

Very Clear: This term describes a fingerprint that exhibits a high degree of clarity and detail. The ridges and galleries are sharply defined, and the majority of the fingerprint’s unique characteristics can be easily identified. A very clear fingerprint would be suitable for high-precision biomedical applications and would present minimal complexity in recognition tasks.

To objectify our assessment, we implemented a standardised scoring system where the surgeons independently evaluated the claim of the fingerprints and compared their findings. In cases with disagreement, a third surgeon was consulted to reach a consensus.

### Temperature sensations

We placed a metal rod into a 4 °C fluid-filled beaker. After 10 min, the metal rod was used to test cold sense. Similarly, we placed another metal rod into a 45 °C fluid-filled beaker. After 10 min, this metal rod was used to test warm sense. When we tested the temperature sensation, one end of the metal rod was directly in contact with the skin. The test was scored by blindfolded candidates themselves. If the candidate had the specified feeling, the result would have been marked as 1. If the candidate had no feelings, the result would have been marked as 0.

### Function of autonomic nerves

The ninhydrin-stained sheets were copied and masked to assess the hidrotic effect [15]. Candidates drank hot water to help sweat. Their fingertips were pressed on the prepared paper. The contour of fingers was portrayed in pencil. A ninhydrin spray was used. The fingertips pressed against paper (quality copy paper) leave a print that can be dyed and fixed with a solution of 1% ninhydrin dissolved in acetone. Sweat spots would show after 24 h. We evaluated and recorded the results. If the sweat had been dyed, the result would have been marked as 1. If the sweat had not been dyed, the result would have been marked as 0.

### Static two-point discrimination

A device consisting of a washer with prongs spaced at various intervals (2, 3, 4, 6, 8, 10, 12, 15, 20, and 25 mm) about its periphery was used to measure static two-point discrimination. Prongs came into contact with the skin from great to small intervals. The test was scored by blindfolded candidates. The minimum discrimination interval was marked if the candidate had the specified feeling. The result was marked 0 if the candidate had no feelings.

### Data processing and statistical analysis

Data were statistically analysed with SPSS 25.0. For continuous variables, the mean and the standard deviation were used to describe our results ($\stackrel{-}{\text{x}}$±s). The normal distribution and variance homogeneity tests were conducted. If the two tests’ results were satisfied, the two independent sample t-tests were used for comparisons between the two groups. One-way ANOVA was used for comparisons amongst multiple groups, and the LSD method was used for inter-group back-testing. Counts (N) and percentages (%) were used to describe summary statistics for categorical variables. The Chi-squared test or the Fisher exact probability method was used to compare group differences. All statistical analyses were two-sided tests. *P* < 0.05 was considered statistically significant.

## Results

### General information

The general data of patients in six groups were compared (Table 1). In the 0–35 days group, there were 25 male cases and 10 female cases with an average age of 36.03 ± 10.95 years (range of 17–53 years). The average size of defects was 7.81 ± 6.86 cm^2^ (range of 1.2–37.5 cm^2^). In the 36–75 days group, there were 13 male cases and 7 female cases with an average age of 38.00 ± 11.10 years (range of 17–55 years). The average size of defects was 9.78 ± 8.22 cm^2^ (range of 2.89–37.5 cm^2^). In the 76–105 days group, there were 5 male cases and 2 female cases with an average age of 40.71 ± 10.39 years (range of 22–51 years). The average size of defects was 9.23 ± 6.70 cm^2^ (range of 4–22 cm^2^). In the 106–215 days group, there were 16 male cases and 3 female cases with an average age of 31.68 ± 11.15 years (range of 17–53 years). The average size of defects was 8.90 ± 4.64 cm^2^ (range of 3.74–22 cm^2^). In the 215–265 days group, there were 11 male cases and 3 female cases with an average age of 33.50 ± 11.55 years (range of 17–53 years). The average size of defects was 6.74 ± 3.36 cm^2^ (range of 1.2–14.4 cm^2^). In the over 365 days group, there were 8 male cases and 8 female cases with an average age of 33.69 ± 13.96 years (range of 15–55 years). The average size of defects was 7.74 ± 3.40 cm^2^ (range of 3.6–14.4 cm^2^). In addition, the preoperative and postoperative conditions of the patient with a defect area of 37.5 cm^2^ are shown in Figure S1.

**Table 1:** General information on patients with free flaps at different follow-up times

	0–35 days	36–75 days	76–105 days	106–215 days	216–356 days	> 365 days
**Sex**						
Male	25	13	5	16	11	8
Female	10	7	2	3	3	8
**Age**	36.03 ± 10.95	38.00 ± 11.10	40.71 ± 10.39	31.68 ± 11.15	33.50 ± 11.55	33.69 ± 13.96
**Position**						
Thumb	6	1	1	4	2	4
Index finger	19	14	5	12	9	10
Middle finger	7	3	1	0	2	1
Ring finger	2	1	0	2	1	1
Little finger	1	1	0	1	0	0
**Size**	7.81 ± 6.86	9.78 ± 8.22	9.23 ± 6.70	8.90 ± 4.64	6.74 ± 3.36	7.74 ± 3.40
**Length of surgery**	215.00 ± 43.90	220.05 ± 48.61	232.43 ± 46.59	212.05 ± 45.77	216.14 ± 35.18	222.88 ± 53.75
**Length of hospital stays**	16.17 ± 4.12	15.95 ± 4.44	29.99 ± 5.29	16.65 ± 5.39	15.36 ± 3.56	15.35 ± 3.76

### Postoperative recovery

All patients were discharged after surgery treatment. No patients were necessary for secondary surgery, and all flaps survived. As shown in Table 2, all five parameters indicated statistical differences amongst the groups. With the increase in time, the recovery degree of fingerprint recognition gradually improved. The degree of recovery at 216 days after surgery was stable at about 75%. Regarding the warm and cold sensations, they also recovered significantly over time. At 1 year after surgery, 3 patients (18.8%) experienced warm intolerance, and 2 patients (15%) experienced cold intolerance; no patient experienced dysesthesia. Two-point discrimination in the 76–105 days group was worse than that in the 36–75 days group. However, as shown in Table 2, we observed that two-point discrimination gradually improved over time, even beyond the postoperative mark of the 36–75 days group (*P* < 0.05). The mean two-point discrimination at 1 year after surgery was 4.25 ± 2.05 mm. No patients had postoperative complications at the donor sites. The proximal interphalangeal mobility loss was less than 10 degrees. All patients returned to their jobs.

**Table 2:** Follow-up outcomes of patients with free flaps at different follow-up times

Group	Fingerprint recognition (%)	Warm Sense(%)	Cold Sense(%)		Two-point discrimination (mm)		Ninhydrin staining(%)		
**0–35 days**	8.57	0		22.86		0 ± 0		0	
**36–75 days**	15	40^a^		55^a^		7.60 ± 4.1^7a^		20^a^	
**76–105 days**	42.86	57.1^a^		71.4^a^		10.86 ± 1.07^ab^		57.14^a^	
**106–215 days**	63.16^ab^	73.7^a^		78.9^a^		7.58 ± 1.43^ac^		78.9^ab^	
**216–365 days**	78.6^ab^	78.6^ab^		85.7^a^		6.14 ± 2.14^ac^		85.7^ab^	
**Over 365 days**	75^ab^	81.2^ab^		87.5^a^		4.25 ± 2.05^abcde^		87.5^ab^	

^a^ Compared to the 0–35 days group, statistical differences are observed, *P*<0.05; ^b^ Compared to the 36–75 days group, statistical differences are observed, *P*<0.05; ^c^ Compared to the 75–105 days group, statistical differences are observed, *P*<0.05; ^d^ Compared to the 106–215 days group, statistical differences are observed, *P*<0.05; ^e^ Compared to the 216–365 days group, statistical differences is observed, *P*<0.05

### Example case

**Fig. 1:**
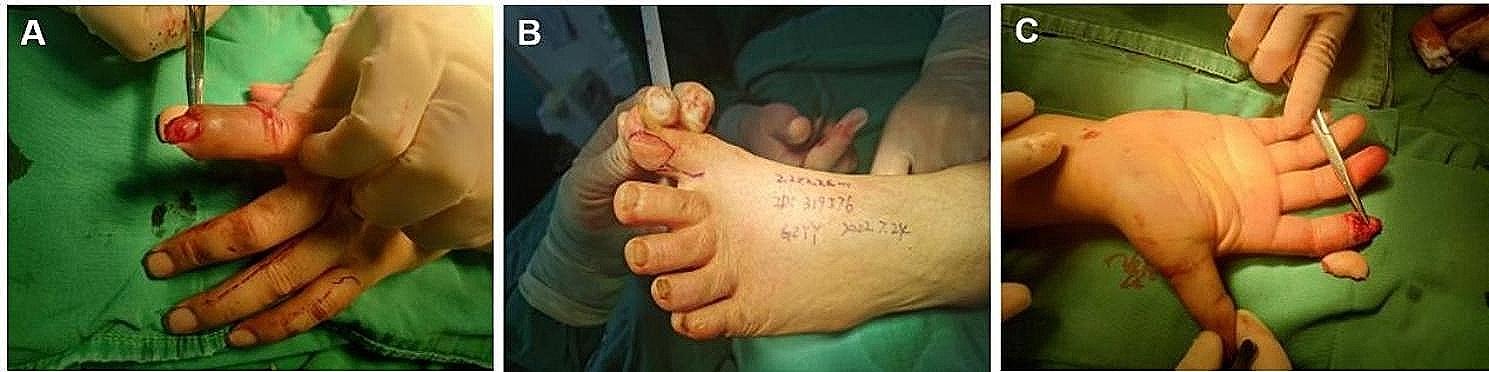
Patient’s finger injury before surgery. **A**, **C**: Preoperative view of soft-tissue defect of distal thumb. **B**: Design of fibular side skin flap of the great toe

**Fig. 2:**
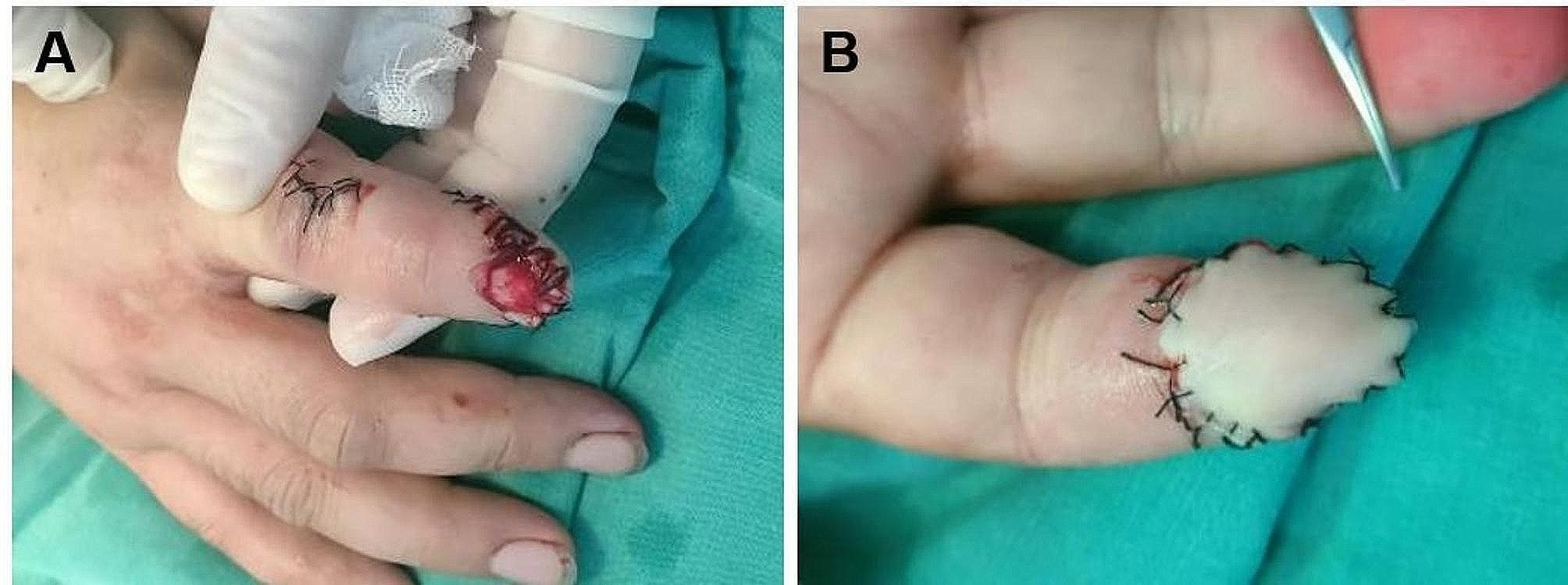
Immediate postoperative view: the defect was reconstructed

**Fig. 3:**
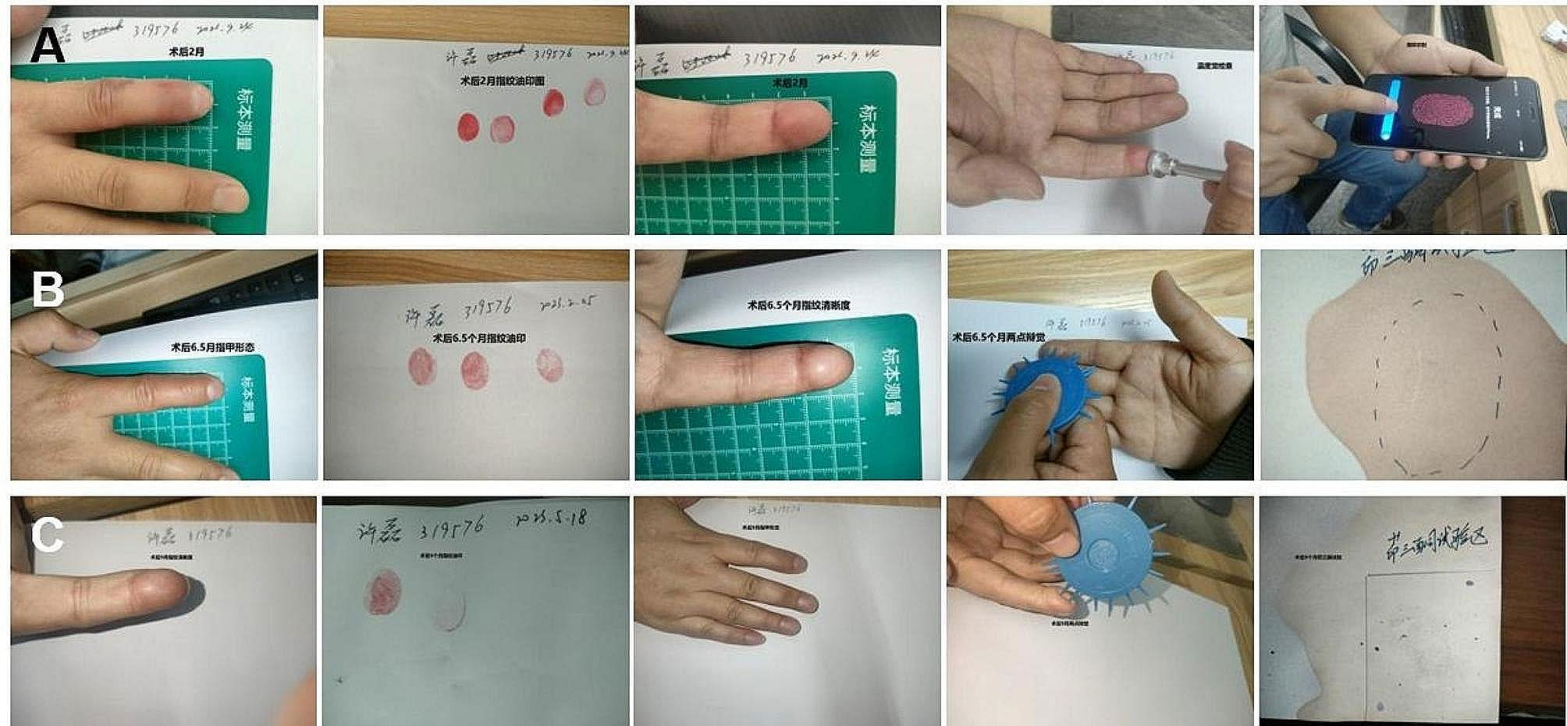
Postoperative recovery of patients. **A**: The function and appearance of the hand site at 2 months after surgery. **B**: The function and appearance of the hand site at 6.5 months after surgery. **C**: The function and appearance of the hand site at 9 months after surgery

A 33-year-old male was admitted for emergency visits because of a crush injury due to a production accident. The finger pulp, nail fold and fingertip of his right distal index finger were the injured parts. The edges of the wound were irregular. A severe contusion of tissue with blood oozing could be observed. The tuberosity of the distal phalanx was partly exposed. The nail bed was still intact. Debridement and free fibular great toe neurovascular flap transplantation were performed after admission. Preoperative photographs of the flap transplantation are shown in Fig. 1. Figure 2 shows the immediate postoperative photographs. The patient required postoperative antibiotics, anticoagulants, antispasmodics, drugs promoting nerve growth activity and heat preservation by visible light illumination. Five days after the initial steps, the patient started rehabilitation training. Two weeks after surgery, stitches were removed, and the patient was discharged. Video 1 shows the postoperative video at 314 days, in which the fingerprint register and recognition process were recorded. Figure 3 shows postoperative photographs at 314 days, in which the appearance and results of the temperature sensation test, the autonomic nerve function test and the static two-point discrimination test were obtained. This patient demonstrated good aesthetic and functional results.

## Discussion

Given the homology of hands and feet, toe flap transplantation is an excellent choice for repairing finger pulp defects [16, 17]. After repair, it has threads and a good appearance [18]. The repaired fingerprint also has biological recognition function, which can be used to unlock various new electronic devices. This study found that the morphology of repaired fingerprints dynamically changes as the postoperative rehabilitation process progresses. During periods 1 to 2, the fingerprint changes from deep to shallow; after the thread loses its innervation, the fingerprint does not immediately disappear and gradually becomes shallow over time. After reaching period 3, the thread gradually deepens due to the recovery of neural function. After period 5, it reaches a stable state without significant changes, so there is a deep shallow process of the thread after surgery. The cold sensation starts from nothing in period 1 and recovers faster as time shifts. The overall recovery time curve of warm sensation is 1 month later than that of cold sensation, and cold sensation is more sensitive than warm sensation. The skin has more cold receptors than heat receptors. Warm sensation gradually recovers in period 4, and its recovery speed and degree are worse than those of cold sensation.

The nerves are mainly sensory and C-type fibres [19]. The phenomenon of skin sweating is a skin reflex potential that occurs after the human body receives stimulation. It originates from the impulses released by the efferent fibres of the sympathetic nervous system, inducing synchronous activity of sweat glands [20]. Therefore, the recovery of the sympathetic nervous system can be detected through the phenomenon of sweating. In this study, we found that sweating had already begun at the beginning of period 2. By period 3, the sympathetic nerves in most of the flaps had recovered to varying degrees, and there was a significant difference compared with the depth of sensation and thread (*P* < 0.05) because the sympathetic nerves affected the regeneration of the surrounding meridians. The use of printing pad oil to record finger touch is a technique that has been practiced by the Chinese for thousands of years. Notably, we found that the fingerprint clarity of oil printing in periods 1, 4, 5 and 6 was the same as that in photos. However, in period 2, the camera’s photo quality and visible clarity were relatively blurry, but the fingerprints pressed out by oil printing were relatively clear, and the specific reasons need to be further studied.

Repaired fingerprints can be applied in modern life [21]. In this study, the use of mobile phone unlocking function was used to record the usage status of fingerprints. During periods 3–5, the success rate of fingerprint unlocking gradually increased. However, the repaired fingerprint shapes in different periods varied, resulting in fingerprints that could be successfully entered in period 2 but unable to be successfully unlocked again in period 3. The fingerprint had to be re-entered to successfully unlock again, and a stable state was reached in periods 4 and 5. This reason was related to the growth and shape of the finger body. As a result of functional needs, the repaired finger body may undergo local tissue remodelling and reconstruction, approaching normal finger growth. In addition, the change in two-point discrimination within different periods could be attributed to several factors: (1) Healing phase: The patients in this time frame of the 76–105 days group might have been in a specific phase of the healing process where tissue maturation and nerve regeneration could temporarily affect sensory acuity, leading to the deterioration in two-point discrimination within the 76–105 days group compared with the 36–75 days group. (2) Longitudinal improvement: Despite the setback observed in 76–105 days, our data indicated that patients experienced a gradual improvement in two-point discrimination with continued rehabilitation and as the healing process progressed. This result was consistent with the general understanding that recovery from nerve injury and sensory restoration is often a protracted process. Therefore, there is a dynamic change in the repaired finger body and fingerprint. The healing between the skin flap and the wound will result in scar formation, which starts from repair and is mainly divided into three consecutive and overlapping stages: the haemostasis/inflammation stage, the granulation tissue proliferation stage and the remodelling stage [22]. This process usually lasts 6–8 months, and the softening and stabilisation process of the subcutaneous scar can lead to changes in the surface thread [23]. 

In this study, some wounds require toenails to repair the nails. After period 4, the appearance and glossiness of most nails were fully restored, which was slower than the normal nail growth cycle. The anastomotic branch from the palmar digital artery to the dorsal digital artery arch and the dorsal digital artery arch from the distal end of the digital artery are the main sources of blood supply for nails [24]. A distal phalanx defect often damages the proper digital artery of the distal phalanx, leading to a decrease in direct blood supply to the nail bed [25]. This phenomenon may also be an important influencing factor for nail bed atrophy and poor glossiness.

Two methods can be used to restore and reconstruct the nerves of the skin flap: the peripheral approach and the central approach [26]. All flaps in this study were directly anastomosed to the central pathway of the sensory nerve, but their peripheral pathways also played a role in the recovery process. The use of the 4-needle epineural anastomosis technique for nerve stumps maximises nerve recovery [27]. The fibular proper artery at the base of the great toe is the main blood supply vessel for the toe flap. It is accompanied with nerves, which can minimise nerve dissociation and ischemia [28]. Therefore, all cases in this study recovered well in terms of personal sensation, and the clarity of fingerprints in the later stage was correlated with nerve recovery.

However, this study had some limitations. Firstly, this work was a single-centre study, and the same surgical team carried out all operations. Follow-up outcomes and surgeons’ skills may be intimately entwined. Secondly, the normal skin on the contralateral side of the recipient area was not tested, and the recovery of the donor sites was not recorded in detail. Lastly, a prospective randomised comparative study with other strategies is needed to yield more meaningful conclusions. In addition, there was a lack of quantitative indicators of photo sharpness. Moreover, the relationship between fingerprint shape and scar, whether there is a relationship between fingerprint shape and the utilisation rate of injured fingers at work, was not systematically studied. Furthermore, given the inconsistent compliance of postoperative patient follow-up, we did not perform dynamic pre- and post-evaluation for each patient at each period, which could have led to a degree of selective bias.

## Conclusion

For the refined reconstruction of the soft-tissue injury in distal fingers, the use of free neurovascular flaps from the fibular side of great toes could achieve satisfactory outcomes.

### Electronic supplementary material

Below is the link to the electronic supplementary material.

Supplementary Material 1

Supplementary Material 2

## Data Availability

The datasets used and analyzed during the current study are available from the corresponding author on reasonable request.
