# Supplementary material for: Repair of distal finger soft-tissue defects with free fibular great toe neurovascular flaps
Source: BMC Musculoskelet Disord. 2024 Jun 18;25:479. doi: 10.1186/s12891-024-07563-2 (PMC11184890; doi:10.1186/s12891-024-07563-2)
Supplement: Supplementary file 2 — Supplementary Material 2 [file 12891_2024_7563_MOESM2_ESM.docx]

**Supplement Information**

**Repair of Distal Finger Soft-tissue Defects with Free Fibular Great Toe Neurovascular Flaps**

Fengnian Yu^a,b†^, Fen Xiao^c†^, Guorui Peng^c^, Gang Lin^c^, Wensong Wang^c^, Chao Xie^b*^, Lijun Lin^b*^

a Department of Orthopedics, Jiangmen People's Hospital, Jiangmen, Guangdong 529020, P. R. China

b Department of Joint and Orthopedics, Zhujiang Hospital, Southern Medical University, Guangzhou, Guangdong 510280, P. R. China

c Department of Orthopedics, Guzhen People's Hospital, Zhongshan, Guangdong 528421, P.R. China

† These authors contributed equally to this study

* Corresponding author: Email: chaoxie1118@foxmail.com; [gost1@smu.edu.cn](mailto:gost1@smu.edu.cn)


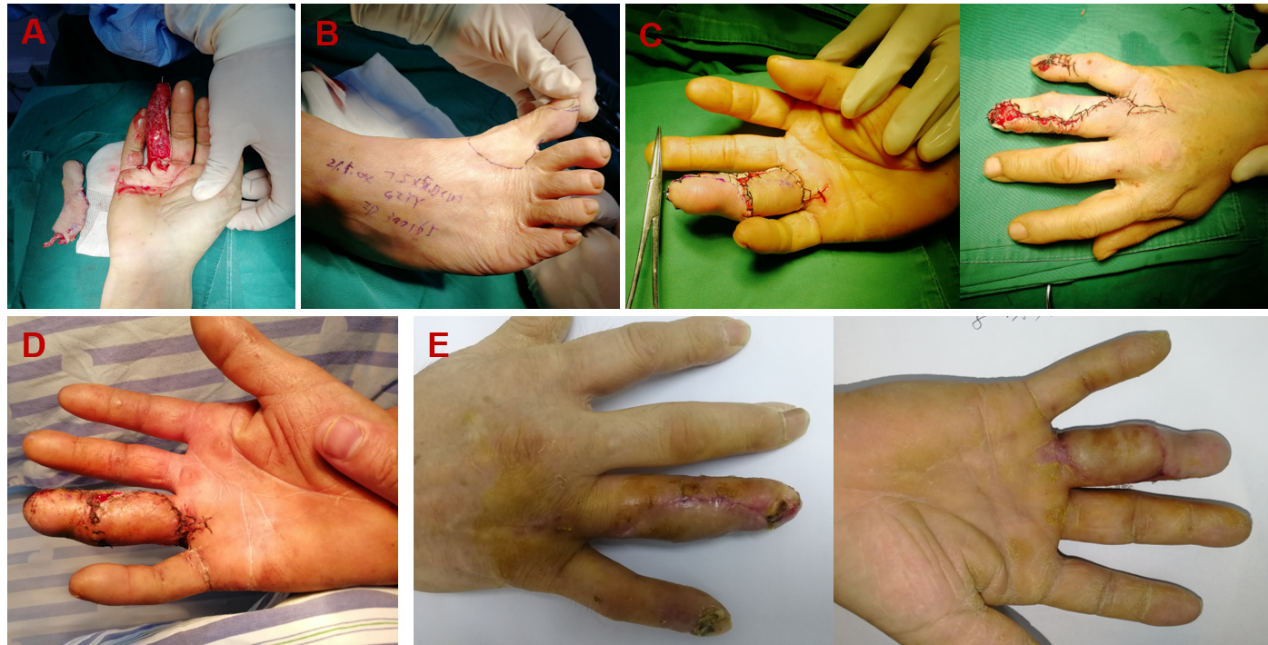


Figure S1. A 47-year-old male was admitted for emergency visits due to a crush injury caused by an avulsion injury of the right ring finger with a skin soft tissue defect. A: Preoperative view of soft-tissue defect of distal thumb. B: Design of fibular side skin flap of the great toe. C: Immediate postoperative view: the defect was reconstructed. D: The recovery of the finger on the 10th day after surgery. E: The recovery of the finger on the 40th day after surgery.
